# Draft Genome Sequence of *Pseudomonas savastanoi* pv. savastanoi Strain DAPP-PG 722, Isolated in Italy from an Olive Plant Affected by Knot Disease

**DOI:** 10.1128/genomeA.00864-14

**Published:** 2014-10-02

**Authors:** Chiaraluce Moretti, Chiara Cortese, Daniel Passos da Silva, Vittorio Venturi, Cayo Ramos, Giuseppe Firrao, Roberto Buonaurio

**Affiliations:** a Dipartimento di Scienze Agrarie, Alimentari e Ambientali, Università degli Studi di Perugia, Perugia, Italy; b International Centre for Genetic Engineering and Biotechnology, Trieste, Italy; c Instituto de Hortofruticultura Subtropical y Mediterránea La Mayora, Universidad de Málaga-Consejo Superior de Investigaciones Científicas (IHSM-UMA-CSIC), Área de Genética, Málaga, Spain; d Dipartimento di Scienze Agrarie e Ambientali, Università degli Studi di Udine, Udine, Italy

## Abstract

Olive knot disease, caused by the bacterium *Pseudomonas savastanoi* pv. savastanoi, seriously affects olive trees in the Mediterranean basin. Here, we report the draft genome sequence of *P. savastanoi* pv. savastanoi DAPP-PG 722, a strain isolated in Italy from an olive plant affected by knot disease.

## GENOME ANNOUNCEMENT

There is an increasing interest in olive (*Olea europaea* L.) growing in many countries, probably due to the benefit of olive oil in human health. Olive knot caused by *Pseudomonas savastanoi* pv. savastanoi represents a serious disease in many olive-producing areas, which can cause a progressive plant decline that leads to reductions in the number of fruit-bearing shoots and in tree yield potential (1). Disease symptoms are characterized by knots on different parts of the plant, mainly on twigs and young branches (2). Many other bacterial species have been reported to be associated with olive knots (3), in particular *Pantoea agglomerans*, *Erwinia oleae* (4), and *Erwinia toletana*. These olive knot associated bacteria have been reported to form a stable interspecies community with *P. savastanoi* pv. savastanoi; to communicate through a quorum-sensing system mediated by *N*-acyl-homoserine lactone signals, and to increase the disease severity when coinoculated with the pathogen in olive plants (5, 6).

Genomics analyses reported to date for *P. savastanoi* pv. savastanoi include only the draft genome sequence of strain NCPPB3335 (7), isolated from an olive knot in France, and the complete plasmid sequence of the three-plasmid complement of this strain (8). We report here the draft genome sequence of *P. savastanoi* pv. savastanoi strain DAPP-PG722, isolated from an olive knot in Perugia (central Italy). Genomic DNA was prepared using the Nextera DNA sample preparation kit (Illumina), according to the manufacturer’s instructions. Sequencing was performed on an IlluminaMiSeq platform using indexed paired-end 250-nucleotide v2 chemistry. The sequencing produced an output of 1,854,337 reads representing approximately 70-fold coverage of the genome. Assembly, made by Edena assembler (9), yielded 412 contigs with a maximum length of 150 kb and an *N*_50_ of 46 kb, assuming a genome size of 6.42 Mb. The G+C content is 57.9%, which is similar to the 57.12% G+C content reported for *P. savastanoi* pv. savastanoi strain NCPPB3335 (7).

Automatic annotation of the genome, performed using RAST (10), predicted a total of 5,972 candidate protein-coding genes in the draft genome sequence of *P. savastanoi* pv. savastanoi DAPP-PG722, with 1,573 of them (35.7%) annotated as hypothetical proteins. This draft genome also contains 57 tRNA and 16 rRNA sequences. A comparative analysis was performed with the genome sequence of *P. savastanoi* pv. savastanoi NCPPB3335 (accession no. CM001834.1) using MUMmer (11). The results showed that 89% of the *P. savastanoi* pv. savastanoi DAPP-PG722 genome aligned with that of NCPPB3335 with an average of 85% of identity.

Several genes encoding ABC transporters for sugars and urea were found exclusively in the DAPP-PG722 genome. Furthermore, it contains genes involved in the biosynthesis of secretion systems I, II, III, IV, and VI. In agreement with data reported for NCPPB3335, the genome of DAPP-PG722 encoded a complete type III secretion system (T3SS). Additionally, a comparison of the effector repertoire of the two strains revealed that they share all 33 T3SS effectors reported for NCPPB3335 (12). However, the DAPP-PG722 genome also encodes the effector gene *hopA1′*, which is absent in NCPPB3335.

### Nucleotide sequence accession numbers.

This whole-genome shotgun project has been deposited at DDBJ/EMBL/GenBank under the accession no. JOJV00000000. The version described in this paper is version JOJV00000000.
